# Reversal of *C9orf72* mutation‐induced transcriptional dysregulation and pathology in cultured human neurons by allele‐specific excision

**DOI:** 10.1002/alz.090215

**Published:** 2025-01-09

**Authors:** Aradhana Sachdev, Kamaljot Gil, Maria Sckaff, Alisha M Birk, Olubankole Aladesuyi Arogundade, Katherine A Brown, Runvir S Chouhan, Patrick Issagholian Lewin, Esha Patel, Kathleen Keough, Bruce Conklin, Alec ST Smith, Claire Clelland

**Affiliations:** ^1^ Gladstone Institutes, San Francisco, CA USA; ^2^ UCSF Weill Institute for Neurosciences, San Francisco, CA USA; ^3^ University of Washington, Seattle, WA USA; ^4^ UCSF Memory & Aging Center, San Francsisco, CA USA

## Abstract

**Background:**

Efforts to genetically reverse C9orf72 pathology have been hampered by our incomplete understanding of the regulation of this complex locus.

**Method:**

We generated five different genomic excisions at the *C9orf72* locus in a patient‐derived iPSC line and a WT line (11 total isogenic lines), and examined gene expression and pathological hallmarks of C9 FTD/ALS in motor neurons differentiated from these lines. Comparing the excisions in these isogenic series removed the confounding effects of different genomic backgrounds and allowed us to probe the effects of specific genomic changes. A coding SNP in the patient cell line allowed us to distinguish transcripts from the normal vs. mutant allele.

**Results:**

Using ddPCR, we determined that transcription from the mutant allele is upregulated at least ten‐fold, and that sense transcription is independently regulated from each allele whereas antisense transcription is regulated by the opposite allele. Surprisingly, excision of the WT allele increased pathologic dipeptide repeat expression from the mutant allele. Importantly, a single allele was sufficient to supply a normal amount of protein, suggesting that the C9orf72 gene is haplo‐sufficient in induced motor neurons. Excision of the mutant repeat expansion reverted all pathology (RNA abnormalities, dipeptide repeat production and TDP‐43 pathology) and improved electrophysiological function, whereas silencing sense expression did not eliminate all DPRs, presumably because of the antisense expression.

**Conclusion:**

These data increase our understanding of C9orf72 gene regulation and inform gene therapy approaches, including ASOs and CRISPR gene editing, and provide a therapeutic CRISPR gene editing approach to treat C9 FTD/ALS.

